# Orthodontic emergencies in a postgraduate clinic: a retrospective analysis

**DOI:** 10.2340/aos.v85.46620

**Published:** 2026-08-17

**Authors:** Vegard B. Vårum, Vaska Vandevska-Radunovic, Helen Pullisaar

**Affiliations:** a Trøndelag County Authority, Steinkjer, Norway; b Department of Orthodontics, Institute of Clinical Dentistry, University of Oslo, Oslo, Norway

**Keywords:** Orthodontic emergencies, full fixed appliance related problems, fixed retainer related problems

## Abstract

**Objective:**

This retrospective study aimed to investigate the frequency, causes, and temporal distribution of orthodontic emergencies at the Department of Orthodontics, University of Oslo.

**Methods:**

All appointments coded as emergencies from 2015 to 2022 were reviewed and manually screened. Emergencies were classified into three categories: (1) during active treatment, (2) during retention, and (3) miscellaneous. A negative binomial generalized linear regression model was used to assess annual and monthly variations. Chi-square tests for independence were applied to analyze relationships between age group and year, gender and year, and reason for emergency. The significance level was set at *p* < 0.05.

**Results:**

Out of 7206 records screened, 3592 met the inclusion criteria, involving 1657 patients – 685 males (41.3%) and 972 females (58.7%). The mean number of emergencies per patient was 2.2, with no significant year-to-year variation in gender distribution. The most common age groups were 10–14 years and over 20 years. Annual emergency numbers ranged from 313 in 2015 to 638 in 2022, with statistically significant variation. Monthly variation was also significant, with July consistently having the fewest emergencies (*p* < 0.001). Most emergencies occurred during active treatment (*n* = 2021), followed by the retention phase (*n* = 1353), and miscellaneous reasons (*n* = 218). The most frequent causes were issues related to full fixed appliances and fixed retainers.

**Conclusions:**

No consistent pattern of orthodontic emergencies was observed, indicating that operator experience, the stage of postgraduate training, and seasonal fluctuations are likely the key influencing factors.

## Introduction

Orthodontic emergencies are unplanned visits typically prompted by mechanical failure, trauma, or pathology, often involving pain, discomfort, or functional impairment. Reports indicate that emergencies comprise a notable proportion of unplanned dental visits, with estimates suggesting that 5–15% of orthodontic appointments are related to emergency care and that approximately 10–30% of patients experience at least one emergency during treatment [1–3].

Mechanical issues constitute the majority of these emergencies, including bracket debonding, archwire or ligature irritation, and appliance breakages [4–6]. Such events may result in mucosal ulceration, discomfort, or impaired appliance function and are often associated with dietary habits, masticatory forces, trauma, or insufficient appliance maintenance [4, 7]. Less commonly, emergencies arise from biological complications, including periodontal irritation, ulceration, or occasional infections [8, 9]. The frequency and nature of emergencies can vary according to patient age, appliance type, compliance, and operator experience [10, 11].

Orthodontic treatment typically spans about 2 years [9], often longer than other dental treatments, which highlights the need for a robust understanding of emergency patterns and their implications for clinical workflow.

Despite the available literature, several knowledge gaps remain. Most studies report short observation periods, limiting the understanding of temporal variation. In addition, previous research has primarily focused on appliance-related breakages, whereas contextual factors such as operator experience, stages of postgraduate training, and patient intake have received little attention. Finally, limited data are available from postgraduate teaching clinics, where case complexity and operator turnover differ from private practice environments. Together, these gaps indicate that the frequency and characteristics of orthodontic emergencies over extended periods and within postgraduate settings are not well understood.

This retrospective study aimed to document the frequency and causes of orthodontic emergencies at the University of Oslo’s Department of Orthodontics from 2015 to 2022 and to analyze their distribution across months and years.

## Methods and materials

### Study design and setting

This retrospective study was conducted from 2015 to 2022 at the Department of Orthodontics, University of Oslo.

### Ethics approval

This study was approved by the Regional Committee for Medical and Health Research Ethics (case no. 527249) and the Data Protection Impact Assessment (case no. 23149933), ensuring proper data handling from dental journal records.

### Participants, inclusion, and exclusion criteria

All patients who sought orthodontic emergency care between 2015 and 2022 were included based on four diagnostic codes: KJE001 (Acute), KJE (Miscellaneous), KJE054 (Lost retainer), and KJE055 (Removal of retainer) (KJE = Internal orthodontic emergency code). Only notes with an urgent/unplanned character regarding mechanical failure, trauma, pain, or concerns related to orthodontic treatment were included. Routine check-ups, non-emergency procedures, record-taking visits, entries from other departments, empty, or duplicate records were excluded.

### Data, categorization, and grouping

Data were extracted from the Salud Dental Suite journal system (version 2018.3.0) at the Department of Orthodontics and securely stored on the TSD (Services for Sensitive Data) platform (p-2274). The dataset was transferred to Microsoft Excel 2016, and information including diagnostic codes, patient gender, and age at the time of emergency was manually recorded by a single author (VBV) for each month and year.

Orthodontic emergencies were categorized as occurring during:

1. Active treatment,
2. Retention phase, or
3. Miscellaneous (A ‘grey area’ including active treatment, retention phase, or interdisciplinary treatment where reasons for visits were more unclear, random, or less urgent).

Patients were grouped by age at the time of the emergency: 0–9 (primary dentition/early mixed dentition), 10–14 (late mixed dentition), 15–19 (permanent dentition), and ≥20 years (adult dentition), according to growth and timing of orthodontic treatment. Annual emergency percentages were calculated relative to total orthodontic appointments.

### Statistical analyses

Statistical analyses were performed using R (version 4.2.2). Negative binomial generalized linear regression was used to assess year- and month-based variations. Chi-square tests were applied for age, gender, and emergency reasons to explore if those categorical values were significantly related to variations by years. A *p*-value < 0.05 was considered statistically significant.

## Results

### General overview

**Figure 1:**
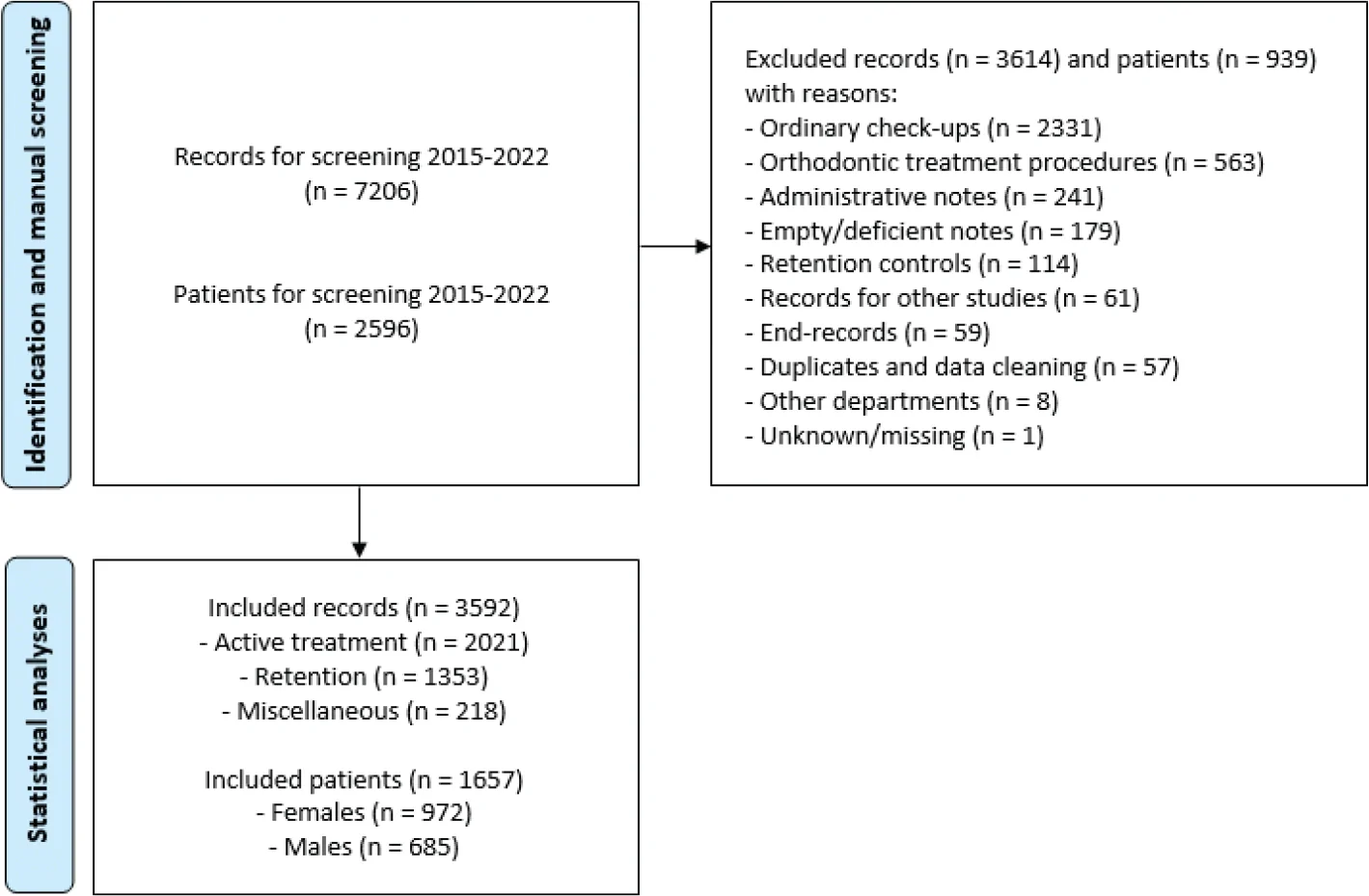
Flowchart illustrating the selection of records and patients.

From 2015 to 2022, a total of 7206 records from 2596 patients were reviewed. Of these, 3592 records from 1657 patients (685 males, 41.3%; 972 females, 58.7%) were included based on emergency criteria. The remaining 3614 records were excluded, out of which 939 patients were completely excluded (see flowchart in Figure 1).

Significant differences for years and months were identified using negative binomial generalized linear regression (Tables 1–3). Although fewer emergencies were recorded in 2020 and 2021, only the reduction in 2021 was statistically significant (*p* < 0.001; Table 1). Significantly more emergencies occurred in 2016, 2017, 2019, 2020, and 2022 than in 2015, 2018, and 2021 (*p* ≤ 0.001; Table 2). July consistently recorded the fewest emergencies (*p* < 0.001; Table 3).

**Table 1:** Emergencies per month and year and their proportion of total appointments.

Month/year	2015	2016	2017	2018	2019	2020	2021	2022	Total month (*n*)
January	35	21	44	49	33	49	29	50	310
February	32	20	49	35	50	40	27	61	314
March	34	29	38	48	50	38	29	83	349
April	27	41	41	34	46	26	27	60	302
May	39	48	31	26	53	30	31	58	316
June	37	50	51	36	42	37	36	53	342
July	15	25	19	21	13	23	17	24	157
August	21	33	36	17	30	31	30	45	243
September	20	66	46	18	43	34	18	58	303
October	19	56	28	19	52	45	27	51	297
November	23	66	53	30	56	46	29	45	348
December	11	43	33	25	63	33	53	50	311
Total year (*N*)	313	498	469	358	531	432	353	638	3592
Total records (*N*)	6056	7877	8079	8081	10,766	8243	8588	10,632	68,322
Percentage (%)	5.2	6.3*	5.8	4.4*	4.9	5.3	4.1*	6.0*	5.3*

A chi-square test was significant (*p < 0.001) for inhomogeneity for percent of emergencies per year. An analysis of standardized Pearson residuals yielded significantly higher than average percentages for the years 2016 and 2022 and significantly lower ones for the years 2018 and 2021 at a Bonferroni-corrected family-wise error rate of 5%.

**Table 2:** Effect of year on annual emergency visit counts.

Year	Estimate	Standard error	*z*-value	Pr(>|*z*|)
2015	-	-	-	-
2016	0.47081	0.10300	4.571	***
2017	0.40447	0.10366	3.902	***
2018	0.13420	0.10683	1.256	0.201
2019	0.52154	0.10251	5.088	***
2020	0.32772	0.10449	3.136	**
2021	0.12529	0.10695	1.171	0.241
2022	0.71312	0.10087	7.070	***

Results obtained by negative binomial regression with log link.

** p < 0.001; *p = 0.001; Base value: 2015.

**Table 3:** Effect of month on monthly emergency visit counts.

Month	Estimate	Standard error	*z*-value	Pr(>|*z*|)
January	0.05384	0.12099	0.445	0.656
February	0.04581	0.12109	0.378	0.705
March	0.14620	0.11981	1.220	0.222
April	-	-	-	-
May	0.05114	0.12102	0.423	0.673
June	0.14029	0.11989	1.170	0.241
July	–0.63790	0.13347	–4.779	**
August	–0.21330	0.12496	–1.707	0.088
September	–0.01466	0.12192	–0.120	0.904
October	–0.02271	0.12203	–0.186	0.852
November	0.14218	0.11986	1.186	0.235
December	0.02870	0.12132	0.237	0.813

Results obtained by negative binomial regression with log link.

** p < 0.001; Base value: April.

### Gender and emergency frequency

Despite a higher proportion of female patients, the annual gender distribution remained consistent (*p* = 0.444, χ² = 6.85, df = 7). The mean number of emergencies per patient was 2.1 for males and 2.2 for females. Nearly half (48.6%) of the patients had only one emergency, and 72.5% had one or two. Thus, the overall median was 2, in good correspondence with mean values, although there were some extreme values. One patient experienced as many as 17 emergencies.

### Age groups

The largest patient groups were aged ≥20 years (*n* = 1320), 10–14 years (*n* = 1276), and 15–19 years (*n* = 945). The 0–9 group had the fewest patients (*n* = 51). Age distribution varied significantly across years (*p* < 0.001, χ² = 74.1, df = 21). Fewer 10–14-year-olds and more ≥ 20-year-olds were seen in 2015 and 2021, while the opposite was observed in 2022.

### Treatment phases

Most emergencies occurred during active treatment (*n* = 2021) or the retention phase (*n* = 1353); few were categorized as miscellaneous (*n* = 218). Active treatment emergencies increased by ~15% in 2019 and 2022, while 2015 recorded the fewest.

### Causes of emergencies

**Figure 2:**
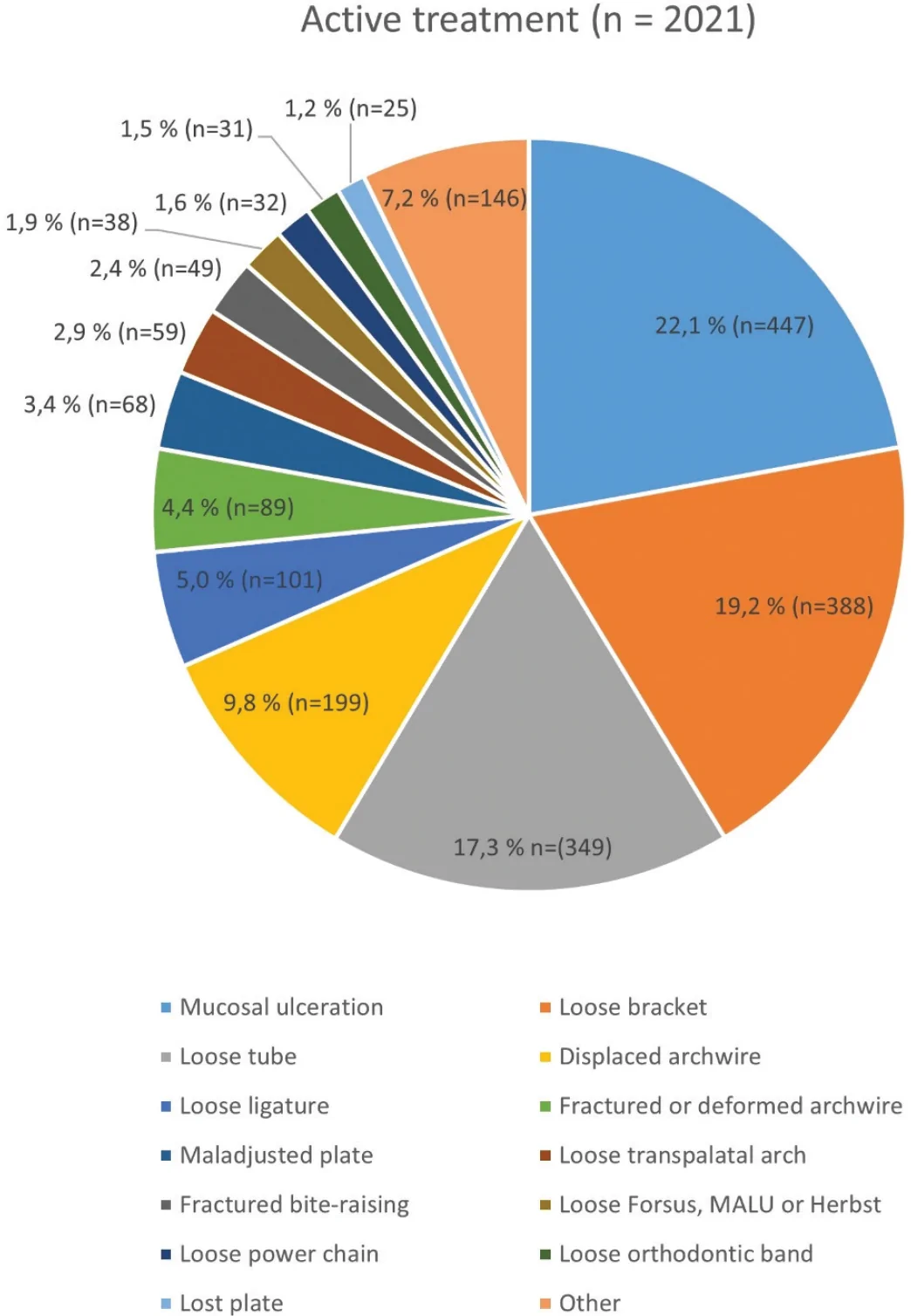
Reasons for emergencies in ‘active treatment’ category. *Other (*n* = 146): Mucosal/gingival changes (*n* = 22), loose or maladjusted Quad Helix (*n* = 17), displaced coil or crimp stops (*n* = 17), problems with orthodontic traction chain (*n* = 16), loose Temporary Anchorage Device(s) (TADs) or pain/swelling (*n* = 13), open bracket (*n* = 10), foreign items in appliance (*n* = 9), loose drop-in-hook (*n* = 8), adverse tooth movement (*n* = 7), fractured aligner attachment (*n* = 6), maladjusted headgear, Delaire mask or lip-bumper (*n* = 5), loose Eva-plate (*n* = 4), loose tongue crib (*n* = 4), loose Rapid Maxillary Expander (RME) (*n* = 4), lost powerarm (*n* = 2), lost lingual arch (*n* = 2).

**Figure 3:**
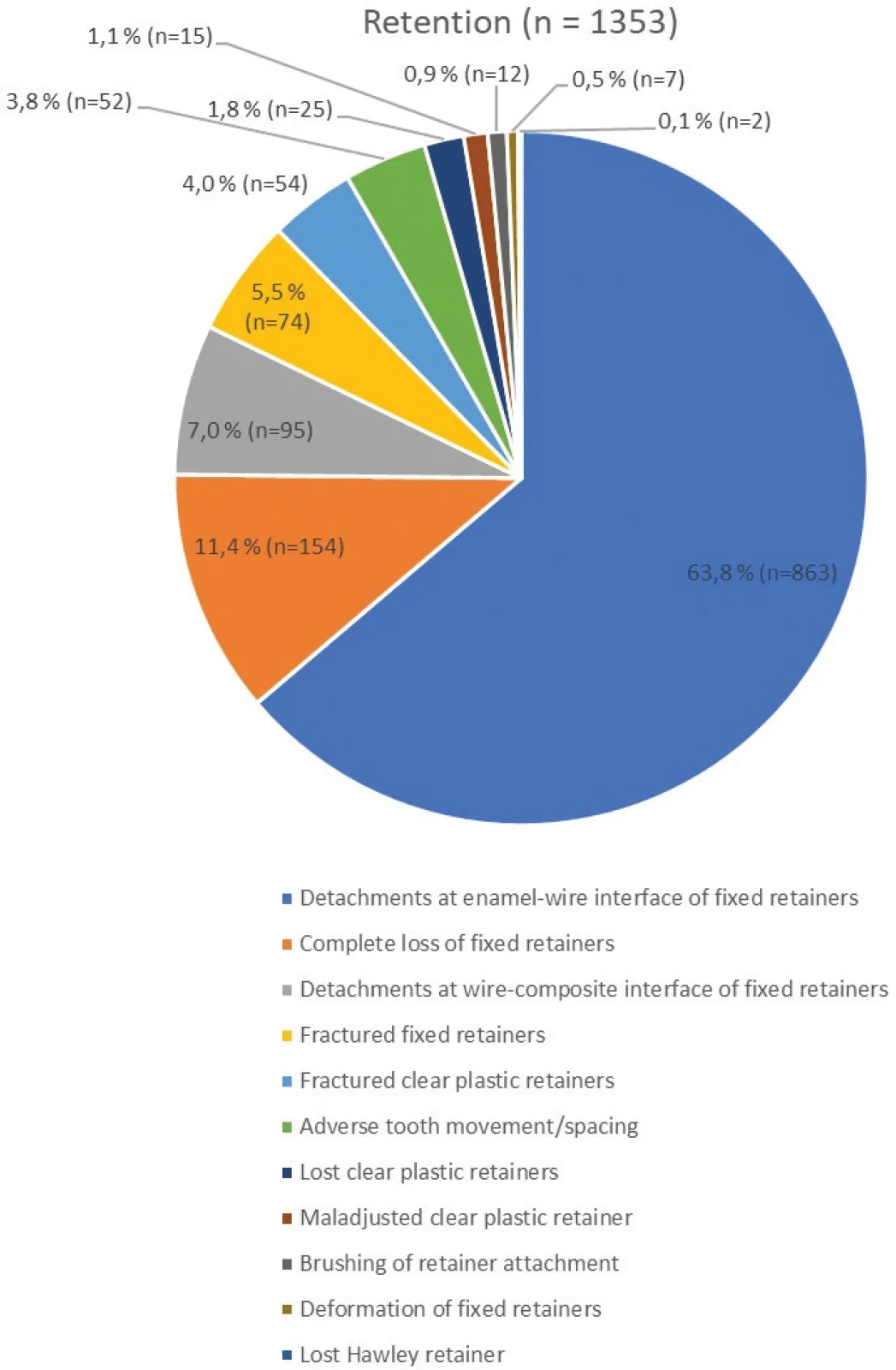
Reasons for emergencies in ‘retention’ category.

**Figure 4:**
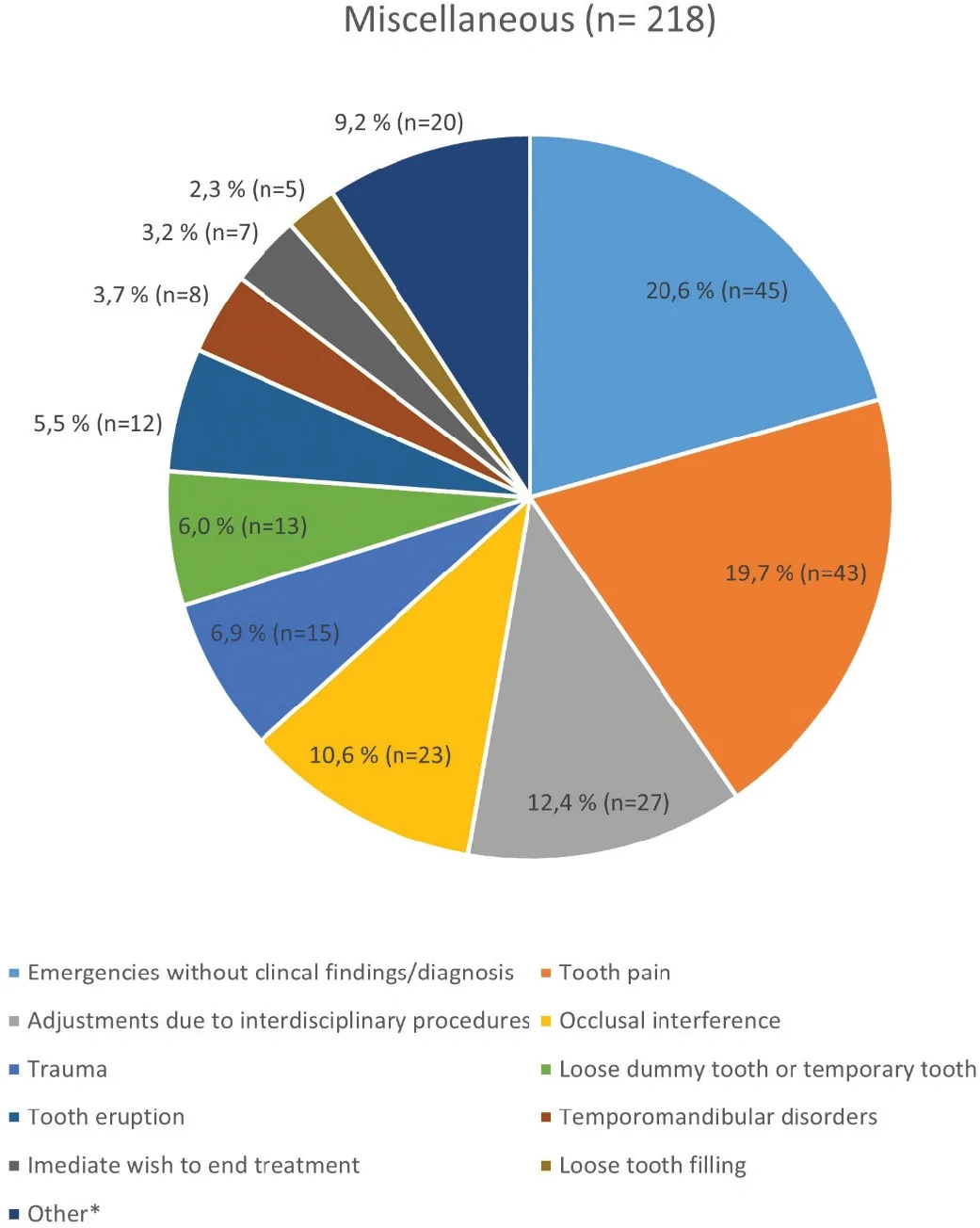
Reasons for emergencies in ‘miscellaneous’ category. *Other (*n* = 20): Enamel fracture (*n* = 2), veneer fracture (*n* = 2), detached Maryland bridge (*n* = 2), rRotational pontic (*n* = 2), tooth mobility (*n* = 2), bonding remains (*n* = 2), removal of sutures (*n* = 1), fractured deciduous tooth (*n* = 1), infraction (*n* = 1), open caries cavity (*n* = 1), loose space maintainer (*n* = 1), root resorption (*n* = 1), lost deciduous teeth when agenesis (*n* = 1), fractured spoon (*n* = 1).

The reasons for seeking emergency care are presented in Figure 2–4. During active treatment, mucosal ulcerations (*n* = 447), typically caused by archwires or ligatures, were the most common reason. Other frequent issues included loose brackets (*n* = 388), loose tubes (*n* = 349), displaced archwires (*n* = 199), fractured/deformed archwires (*n* = 89), and loose ligatures (*n* = 101).

During the retention phase, problems related to fixed retainers were the most frequent, particularly detachments at the enamel-wire interface (*n* = 863), detachments at the wire-composite interface (*n* = 95), and fractured fixed retainers (*n* = 74). Deformation of fixed retainers (*n* = 7) was less common, whereas complete loss of fixed retainers (*n* = 154) occurred more frequently. In contrast, removable retainers caused fewer emergencies, which included fractured (*n* = 54) or lost (*n* = 25) clear plastic retainers, as well as lost Hawley retainers (*n* = 2). Adverse tooth movement/spacing (*n* = 52) was another common reason for seeking care during retention.

The ‘Miscellaneous’ category encompassed a variety of less frequent issues. The most common emergencies in this category were emergencies without clinical findings/diagnosis (*n* = 45), adjustments of orthodontic appliances due to interdisciplinary procedures (*n* = 27), and tooth pain (*n* = 43).

## Discussion

This retrospective study demonstrates a rather similar annual distribution of orthodontic emergency appointments at a postgraduate orthodontic clinic. In 2020, a non-significant decrease in emergencies occurred, which continued in 2021 and then showed a statistically significant reduction compared to the rest of the investigated period. Emergency cases as a percentage of total appointments showed slight annual variation, with July consistently recording the fewest emergencies.

The sample had more female patients than male, but emergencies were evenly distributed between genders. Most patients were adults and adolescents, with emergencies more common during active treatment with fixed appliances, mainly due to displaced wires or detached brackets.

The proportion of emergencies relative to total annual clinical activity ranged from 4.1 to 6.0%. Although the year-to-year variations are statistically significant, they are relatively minor, suggesting that emergency cases represent a consistent proportion of costs, service demands, and resource allocation. Variations in the annual number of emergencies can be attributed to multiple factors, with operator experience being particularly influential. New cohorts of postgraduate orthodontic students were admitted every third year, in 2015, 2018, and 2021, while peaks in emergencies were observed the next years, 2016, 2019, and 2022. Peaks of emergencies coincided with early stages of training. While research specifically addressing orthodontic treatment is limited, previous studies assessing operator-related factors in bonding procedures [10] and endodontic treatments [11] have demonstrated the critical role of experience.

The number of patients in active treatment was higher in 2020 than in 2021. This may be attributed to the strict lockdown measures implemented at the onset of the COVID-19 pandemic, potentially delaying recruitment of new patients. A British study found that orthodontic referrals from patients in the lowest socioeconomic groups declined by 5.27% at the onset of the pandemic, followed by recovery over the subsequent year [12]. A possible reduction in referrals, coupled with a transition period for the incoming postgraduate students in 2021, may have contributed to the lower number of patients in active treatment and, consequently, to the decline in emergencies observed during that year.

Emergency patterns also followed seasonal trends. July consistently had fewer emergencies, likely due to holidays and reduced clinic hours, with increases before and after suggesting a ‘bottleneck’ effect. This may reflect both practical and psychological scheduling factors.

The sample in this study comprised 685 males (41.3%) and 972 females (58.7%), a distribution consistent with findings from other studies investigating orthodontic emergencies [13] and monthly new appointments [14] during the COVID-19 pandemic. Another study evaluating the uptake of patients for orthodontic treatment reported an even higher proportion of female patients [15]. In general, it may be hypothesized that women utilize health services more frequently than men. Statistics confirm this trend in the Norwegian healthcare utilization, with the average number of medical appointments in 2022 being 3.6 for women and 2.4 for men [16]. However, this gender difference does not extend to acute medical care appointments, which are more evenly distributed between women and men, averaging 0.25 and 0.22 visits, respectively [16]. The minimal difference in the number of emergencies between genders observed in our study suggests that both men and women seek emergency care when necessary.

Our study showed that most emergencies occurred with fixed appliances during active treatment. The reasons for seeking orthodontic emergency care were consistent with those reported in other studies. Al-Fadhily et al. [13] identified broken or debonded brackets (55.6%) and long pocking wires (35.2%) as the most frequent causes for orthodontic emergency visits. Miao et al. [17] reported that debonded brackets (50%), pocking wires or sharp ligature ties (44.4%), and ulcers (19.4%) were the most common issues among patients with fixed appliances. Furthermore, attachment detachments (50%), depletion of aligners (43.8%), shortage of elastic bands (18.8%), and difficulties in positioning aligners (18.8%) were the predominant issues reported among patients using clear plastic aligners [17]. Emergencies related to fixed appliances were associated with higher levels of pain and functional impairment compared to those involving clear plastic aligners [17].

Problems related to fixed retainers in the retention phase were also a common cause of emergency visits. Retainer detachment due to composite failure is frequently reported in the litterature [18–20], whereas fractures and complete loss occur less frequently [18]. The survival rate of fixed retainers varies from 40 to 75% [18, 20–22], depending on material, location (maxilla or mandible), maintenance, and duration of follow-up. In our study, 155 patients attended five or more appointments during the retention phase. Fixed retainers accounted for a substantial proportion of repeated appointments, highlighting the importance of individual factors. The difference in emergency frequency between fixed and removable retainers is likely attributable to variations in the retention protocols. Fixed retainers are often routinely bonded to both jaws, whereas removable retainers are typically fabricated only for the maxilla [23, 24].

The low number of emergencies in the 0–9-group may be related to the stage of tooth development. At this age, most patients receive removable appliances with shorter treatment durations, which appear to result in fewer adverse effects. In this study, 61.2% of the appointments involved patients aged 10–14 and 15–19 years. The decrease in patients aged 10–14 years in 2021 is somewhat unexpected and may be attributed to a combination of factors, including delayed effects of COVID-19 restrictions, a high rate of treatment completions before summer holidays, and fewer treatment initiations that year.

### Limitations

The retrospective design of this study presents several limitations. Incomplete or inaccurate records may have introduced bias in data interpretation, which was conducted by a single examiner. Approximately 15% of visits recorded two reasons for emergency care, while fewer than 1% recorded three. To minimize overestimation, overlapping codes were not accounted for; however, this approach may have resulted in underestimation of certain categories.

### Generalizability

The dataset was not adjusted for socioeconomic status or health conditions. The study was conducted at a postgraduate clinic, which may limit the generalizability of the findings.

Distribution of appliances in the study population was not a part of the original data set. At the investigated periods, there were very few aligner patients in the postgraduate clinic. The department runs internal self-assessment of patient treatments, and according to our last data, 93% of the patients are treated with fixed appliances. The rest are treated with removable appliances but mainly as an introductory treatment, which means that they will most probably get fixed appliances at a later stage. Distribution of appliances affects external validity of the study results, as aligner treatment is more common in private orthodontic clinics.

## Conclusions

Between 2015 and 2022, the frequency of emergency visits at the postgraduate orthodontic clinic at the University of Oslo fluctuated from year to year, with no consistent pattern emerging.

The primary contributor to emergency incidence was seasonal trends, while operator experience and the stages of postgraduate training may be contributing factors. Most emergencies were associated with fixed orthodontic appliances and fixed retainers, which were the most frequently reported causes.

## Data Availability

Date available on request due to privacy/ethical restrictions.
